# Human adipose-derived stem cell transplantation as a potential therapy for collagen VI-related congenital muscular dystrophy

**DOI:** 10.1186/scrt411

**Published:** 2014-02-12

**Authors:** Vitali Alexeev, Machiko Arita, Adele Donahue, Paolo Bonaldo, Mon-Li Chu, Olga Igoucheva

**Affiliations:** 1 Department of Dermatology and Cutaneous Biology, Jefferson Medical College, Thomas Jefferson University, BLSB, Rm. 430, 233 South 10th Street, Philadelphia, PA 19107, USA; 2 Departments of Biomedical Sciences, University of Padova, Padova 35131, Italy

## Abstract

**Introduction:**

Congenital muscular dystrophies (CMD) are a clinically and genetically heterogeneous group of neuromuscular disorders characterized by muscle weakness within the first two years of life. Collagen VI-related muscle disorders have recently emerged as one of the most common types of CMD. COL6 CMD is caused by deficiency and/or dysfunction of extracellular matrix (ECM) protein collagen VI. Currently, there is no specific treatment for this disabling and life-threatening disease. The primary cellular targets for collagen VI CMD therapy are fibroblasts in muscle, tendon and skin, as opposed to muscle cells for other types of muscular dystrophies. However, recent advances in stem cell research have raised the possibility that use of adult stem cells may provide dramatic new therapies for treatment of COL6 CMD.

**Methods:**

Here, we developed a procedure for isolation of human stem cells from the adipose layer of neonatal skin. The adipose-derived stem cells (ADSC) were examined for expression of ECM and related genes using gene expression array analysis. The therapeutic potential of ADSC was assessed after a single intramuscular transplantation in collagen VI-deficient mice.

**Results:**

Analysis of primary cultures confirmed that established ADSC represent a morphologically homogenous population with phenotypic and functional features of adult mesenchymal stem cells. A comprehensive gene expression analysis showed that ADSC express a vast array of ECM genes. Importantly, it was observed that ADSC synthesize and secrete all three collagen VI chains, suggesting suitability of ADSC for COL6 CMD treatment. Furthermore, we have found that a single intramuscular transplantation of ADSC into *Col6a1*^*−/−*^*Rag1*^*−/−*^ mice under physiological and cardiotoxin-induced injury/regeneration conditions results in efficient engraftment and migration of stem cells within the skeletal muscle. Importantly, we showed that ADSC can survive long-term and continuously secrete the therapeutic collagen VI protein missing in the mutant mice.

**Conclusions:**

Overall, our findings suggest that stem cell therapy can potentially provide a new avenue for the treatment of COL6 CMD and other muscular disorders and injuries.

## Introduction

Knowledge of the genetic and molecular mechanisms underlying congenital muscular dystrophies (CMDs) has dramatically advanced in the past decade [1]. However, treatment options for CMDs have remained limited and there is no cure for this group of disabling and often lethal disorders. The CMDs present with muscle pathologies similar to those seen in traditional muscular dystrophies, of which Duchenne and Becker muscular dystrophies are the major forms. However, the mechanisms leading to the muscle pathologies (sarcolemma instability, degeneration and regeneration of muscle cells, apoptosis and fibrosis) differ between the common CMD types and other muscular dystrophies. Gene mutations that result in disturbed interactions between extracellular matrix (ECM) and muscle cells underlie the most prevalent CMD types, that is COL6 CMD, LAMA2 CMD or MCD1A, and various forms of α-dystroglycanopathies [2].

COL6 CMD is the most or the second most common CMD type in the North American, Japanese and Northern England populations [3-5]. Disease is characterized by muscle weakness during the first two years of life [1]. Ullrich congenital muscular dystrophy (UCMD) and Bethlem myopathy, respectively, represent the severe and mild end of a clinical continuum associated with a deficiency or dysfunction of collagen type VI [1,6]. Patients afflicted with COL6 CMD manifest not only muscle weakness but also connective tissue abnormalities, including joint contractures and distal hypermobility. Severely affected UCMD patients are never able to walk independently and suffer from respiratory failure, resulting in early death. The disease is caused by dominant or recessive mutations in the genes encoding collagen VI subunits [1]. Collagen VI is produced by diverse connective tissue cell types in almost all organs. In the skeletal muscle, collagen VI is synthesized by muscle fibroblasts but not by muscle cells [7,8]. The protein is composed of different subunits and the most common form is made up of α1(VI), α2(VI) and α3(VI) collagen chains, encoded by the *COL6A1*, *COL6A2* and *COL6A3* genes, respectively [9]. The severe UCMD phenotype is caused by either recessive or dominant negative mutations in any of the three collagen VI genes [1]. The recessive UCMD patients typically have nonsense or frameshift mutations, resulting in a complete absence or drastic reduction of the collagen VI protein [10-12]. In COL6 CMD, the proteins at fault reside outside of the muscle cells, which is in stark contrast to most other muscular dystrophies, in which the gene mutations usually involve cellular proteins produced by muscle cells. Thus, even though several therapeutic approaches have been explored for traditional muscular dystrophies, there is a need to develop treatment strategies that specifically target muscle ECM alterations.

A mouse mutant lacking the α1(VI) collagen chain, the *Col6a1*^−/−^ mouse generated by gene targeting, has served as a model for recessive UCMD [13]. Like the mouse model for Duchenne muscular dystrophy, the phenotype of the *Col6a1*^*−/−*^ mice is significantly milder than that of the recessive UCMD patients with a total absence of collagen VI protein. The *Col6a1*^*−/−*^ mice have normal life spans and their skeletal muscles display relatively mild myopathic pathology, including variation in muscle fiber diameter and the presence of muscle fibers with centrally localized nuclei. Physiological assessment of the muscles has demonstrated a loss of contractile strength, in that tetanic and twitch tensions were significantly reduced. Myoblasts from the *Col6a1*^*−/−*^ mice and UCMD patients have been shown to display a latent mitochondrial dysfunction that predisposes muscle fibers to apoptosis [14,15]. The abnormality in mitochondria and increase in apoptosis have recently been demonstrated to result from defective autophagy [16]. This has led to a pilot treatment (one month) and a long-term trial (1 to 3.2 years) of a small number of COL6 CMD patients in Italy, using oral administration of cyclosporine A to inhibit the mitochondrial dysfunction [17,18]. The long-term cyclosporine A treatment was shown to be well tolerated and was able to ameliorate performance in limb muscles, but not in respiratory muscles. The results suggest that the pharmacological treatment, although promising, is not sufficient for treating the disease.

Stem cell-based therapy holds promise for treating genetic diseases and has been utilized in animal models and human clinical trials for different types of muscular dystrophies, in particular Duchenne muscular dystrophy [19]. The majority of these studies evaluated the ability of muscle-derived progenitor or stem cells as well as adult stem cells from non-muscle tissues to replace damaged muscle fibers. However, effective cellular therapy for ECM-related CMDs rests on the ability of the therapeutic cells to secrete normal ECM proteins that can prevent muscle cell degeneration rather than on the potential of these cells to differentiate into muscle fibers. Multipotent mesenchymal stem cells (MSC) were originally isolated from bone marrow and subsequently from many other adult tissues. MSC display extensive proliferation capacity in culture and can differentiate *in vitro* into various connective tissue cell types [20,21]. These unique features make MSC an attractive therapeutic option for COL6 CMD. Although bone marrow is the main source for MSC isolation, subcutaneous fat represents an alternative repository for stem cells and is currently a subject of intensive investigations [22]. Similarly to bone marrow-derived MSC, adipose-derived stem cells (ADSC) can be induced to differentiate into multiple lineages *in vitro* under adipogenic, osteogenic, chondrogenic, myogenic and neurogenic supplementation conditions [23]. Furthermore, ADSC can be obtained by less invasive methods, have relatively lower donor-site morbidity and are available in large quantities for procurement [24]. Also, ADSC have immunoprivileged behavior in immunocompetent mouse models, suggesting their potential as immunopriviledged universal cells with the capacity to be used in the allogeneic setting and to reduce graft-versus-host disease [24].

In this study, we developed a procedure for the isolation of ADSC from human neonatal foreskin and demonstrated that the established primary cultures represent a cell population with phenotypic and functional features of mesenchymal progenitors. We also showed that ADSC cultured *in vitro* secrete a variety of ECM proteins, including collagen VI and, therefore, can provide therapeutic ECM proteins without cell differentiation in the muscle environment. In addition, we explored for the first time the potential of xenogeneic ADSC in treating COL6 CMD using *Col6a1*^*−/−*^ mice, the animal model for recessive UCMD. Our results show that ADSC delivered intramuscularly are engrafted in the interstitial connective tissues of the skeletal muscle where they continued to secrete collagen VI and assemble collagen VI microfibrils. The number of collagen VI positive myofibers increased steadily over time after transplantation. The extent of ADSC engraftment and collagen VI production was significantly higher when muscle was injured by cardiotoxin. Our study provides the proof-of-concept evidence that ADSC therapy has the potential to treat COL6 CMD.

## Methods

### Mouse strains

The *Col6a1*^*−/−*^ mice have been described previously [13]. Immunocompromised *Col6a1*^−/−^ mice were generated by crossing the *Col6a1*^*−/−*^ and *Rag1*^*−/−*^ (B6.129S7-*Rag*^*1tm1Mom*^/J, Jackson Laboratory, Bar Harbor, ME, USA) mice. The *Col6a1*^*−/−*^*Rag1*^*−/−*^ genotype was selected by PCR. *Col6a1*^*−/−*^*Rag1*^*−/−*^ mice were used in all ADSC transplantation studies to avoid possible rejection of human cells.

### Isolation of adipose-derived stem cells from human neonatal foreskin and tissue culture conditions

Human adipose-derived stem cells were isolated from discarded neonatal foreskin. The study was not classified as human subject research as per guidelines of the Thomas Jefferson University IRB and as such did not require ethics approval. The use of discarded tissue for research purpose does not require patient informed consent, the hospital’s general informed consent for surgery/procedures specifies that the removed tissue may be collected anonymously and used for research. Briefly, tissue samples were washed two times in PBS plus 1% Penicillin/Streptomycin (Gibco, Grand Island, NY, USA) and then the adipose tissue was separated from the connective tissue using a surgical blade. The tissue was minced and digested in collagenase solution (0.001 g collagenase I (Sigma, St. Louis, MO, USA) in 1 ml PBS and BSA). To obtain a single cell suspension, the digested tissue was applied to a 30 μm mesh separation filter (Miltenyi Biotec, Auburn, CA, USA). A PBS plus 1% BSA solution was added to the mesh to quench the enzyme and flush any remaining cells through the filter. The suspension was centrifuged and the pellet was resuspended in 1 ml of (D)MEM/F12 and GlutaMax™ plus 10% fetal bovine serum (FBS) (Gibco). Cells were plated in (D)MEM/F12 and GlutaMax™ plus 10% FBS (Invitrogen, Grand Island, NY, USA) and grown to confluence. To remove hematopoietic cells, cells were then depleted using magnetic separation beads. For immunodepletion, the cell pellet was resuspended in 40 μl PBS plus 0.5% BSA plus 2 mM ethylenediaminetetraacetic acid (EDTA) (depletion buffer), then 20 μl of CD45 microbeads (Miltenyi Biotec), 20 μl of FcR blocking reagent (Miltenyi Biotec) and 20 μl of CD31 microbeads (Miltenyi Biotec) were added. The cells were incubated on ice for 15 minutes. The cells were washed with depletion buffer and centrifuged. The cell pellet was resuspended in depletion buffer and applied to the MiniMACS magnetic field column system (Miltenyi Biotec) using the MACS MS separation column (Miltenyi Biotec). The eluted cells were collected after several washes and centrifugation. The collected cells were designated as first passage cells and were cultured in (D)MEM/F12 and GlutaMax™ plus 10% FBS at 37°C in a humidified 5% CO_2_-95% air atmosphere.

### Fluorescence-activated cell sorting analysis

ADSC were grown until confluent, trypsinized and pelleted by centrifugation at 200 g for five minutes. For fluorescence-activated cell sorting (FACS) analysis, approximately 2.5 × 10^5^ cells were resuspended in 100 μl FACS buffer containing 10% FBS in PBS. For FACS analysis of surface markers, each sample was incubated for 30 minutes at 4°C with fluorescein isothiocyanate (FITC)- or phycoerythrin (PE)-conjugated antibodies against the following surface markers: CD45, CD34, CD11b, CD31, CD19, CD90, CD44, CD71, CD29, CD73, STRO-1, HLA-ABC, HLA-DR, CD117, CD105, CD106 (eBioscience, San Diego, CA, USA) according to the manufacturer’s instructions. After incubation, the labeled cells were diluted with 2 ml of FACS buffer, pelleted and resuspended in 500 μl of FACS buffer. Generally, approximately 10^4^ cells were analyzed per sample using the BD FACSCalibur flow cytometer (BD Biosciences, San Jose, CA, USA). Results were analyzed using FlowJo software.

### Differentiation assays

Before addition of inductive media, ADSC cultures were grown to confluence after which the standard adipose stem cell medium was replaced with the inductive media. Osteogenic differentiation was induced by culturing cells with osteoinductive medium (Lifeline Cell Technology, Frederick, MD, USA). The cells were treated with osteogenic supplements for up to five weeks with three medium changes per week. Cells were morphologically examined and assessed for alkaline phosphatase (ALP) activity and ECM calcification. Analysis of ALP activity was done using the ALP Semiquantitative Histochemical Diagnostic Kit (Sigma). For matrix mineralization, three-week osteogenic cultures were fixed with 100% ethanol and analyzed using 2% Alizarin Red Stain (Lifeline Cell Technology). Cultures were also analyzed for mRNA transcripts of *ALP* and *BGLAP/OCN*, which denote markers of a mature osteoblast. Total RNA was isolated as described below and subjected to RT–PCR amplification using gene-specific primers listed in Additional file 1: Table S1.

To induce adipocytic differentiation, cells were cultured in adipogenic induction medium (Lifeline Cell Technology) for five weeks with three medium changes per week. For histochemical examination, adipogenic cultures were stained for the presence of intracellular lipid droplets using Oil Red-O staining as an indicator of intracellular lipid accumulation. Briefly, cells were fixed for 30 minutes in 4% paraformaldehyde (Lifeline Cell Technology), washed with 100% 1,2-propanediol dehydration solution (Lifeline Cell Technology) and then stained for 30 minutes at 37°C with 0.5% Oil Red-O solution (Lifeline Cell Technology). Adipogenic cultures were also analyzed for mRNA transcripts of *LPL*, expressed in preadipocytic as well as adipocytic cells and *PPARγ2*, a transcription factor expressed in mature adipocytes. The gene-specific primers are listed in Additional file 1: Table S1.

Chondrogenic differentiation was performed using the micromass culture technique. Briefly, 10 ml of a concentrated cell suspension, at least 1 × 10^6^ cells, were plated into the center of each well of a 96-well plate and allowed to attach at 37°C for two hours. Chondrogenic medium (Cambrex, East Rutherford, NJ, USA) was gently overlaid so as not to detach the cells nodules. Cultures were maintained in chondrogenic medium for up to five weeks with three medium changes per week. Sulfated proteoglycans were specifically detected using Alcian blue staining under acidic conditions. Briefly, cell nodules were fixed with 10% formalin for 20 minutes and washed extensively with PBS. Fixed cells were then incubated for 30 minutes with 1% (w/v) Alcian blue (Sigma) in 0.1 M HCl at pH 1.0 and washed with 0.1 M HCl for five minutes to remove excess stain. Chondrogenic cultures were also analyzed for mRNA transcripts of *SOX9, COLII, COLX, COLXI and ACAN/AGN*. Gene-specific primers are listed in Additional file 1: Table S1.

### Gene expression analysis of stem cells

Total RNA from ADSC was isolated using the Qiagen RNeasy Mini Kit (Qiagen, Valencia, CA, USA). A whole human genome oligo microarray (one-color) (Agilent Technologies, Santa Clara, CA, USA) was performed by a service provided by Miltenyi Biotec. Briefly, the RNA was amplified and labeled with Cy3 using the Agilent Low Input Quick Amp Labeling Kit (Agilent Technologies) according to the manufacturer’s protocol. The hybridization was performed according to the Agilent 60-mer oligo microarray processing protocol using the Agilent Gene Expression Hybridization Kit (Agilent Technologies). All steps including amplification, labeling and hybridization were performed as a service by Miltenyi Biotec. Fluorescence signals of the hybridized Agilent Microarrays were detected using Agilent’s Microarray Scanner System (Agilent Technologies). The differential gene expression data were analyzed using the Rosetta Resolver® gene expression data analysis system (Rosetta Biosoftware). The complete microarray data are available at the National Center for Biotechnology Information (NCBI) Gene Expression Omnibus (GEO) repository under Series Accession Number GSE51030.

### RNA isolation and RT–PCR analysis

Total RNA was isolated using the Qiagen RNeasy Mini Kit (Qiagen). RT–PCR was conducted using the Verso 1-Step RT-PCR ReddyMix Kit (Thermo Scientific, Waltham, MA, USA). For a standard reaction, 50 ng of total RNA was mixed together with 200 nM each of primers in a final volume of 25 μl. All primers and melting temperatures (Tm) used in the study are listed in Additional file 1: Table S1. The amplification cycles for PCR were 50°C for 15 minutes, 95°C for 2 minutes, followed by 35 cycles of 95°C for 20 seconds, 50 to 60°C for 30 seconds and 72°C for 1 minute, followed by an additional extension for 5 minutes at 72°C. The amplified fragments were separated on 1.5% agarose gel in 1 × Tris-acetate EDTA (TAE) buffer at 100 V for one hour and visualized with ethidium bromide staining on a GelDoc 1000 (BioRad, Hercules, CA, USA).

### Western blot analysis

Denatured proteins were isolated from ADSC. Proteins were separated by a 4% to 12% gradient SDS-polyacrylamide gel electrophoresis (PAGE) and transferred to a polyvinylidene difluoride (PVDF) membrane followed by the Western blot analysis using polyclonal anti-COL6A1 [25], anti-COL6A2 [26] and anti-COL6A3 antibodies [27]. Immunocomplexes were detected by using horseradish peroxidase (HRP)-labeled anti-rabbit secondary antibodies (Promega, Madison, WI, USA) and visualized by using SuperSignal WestFemto substrates (Pierce, Rockford, IL, USA).

### Indirect immunofluorescent analysis

For indirect immunofluorescent analysis, cells cultured in chamber slides (Nalgen/Nunc, Rochester, NY, USA) were fixed with methanol at -20°C and blocked with 1% BSA in PBS for one hour at room temperature. Proteins were detected with primary antibodies generated against collagen VI α1, α2 and α3 chains (1:1000 dilutions), respectively, for one hour at room temperature. Immunocomplexes were detected with AlexaFluor^488^- or AlexaFluor^594^-conjugated secondary antibodies (Invitrogen) at a dilution of 1:200 for one hour incubation at room temperature. To visualize nuclei, slides were counterstained with 4′,6-diamidino-2-phenyl indol (DAPI; 1:1000 dilution) for two minutes at room temperature. Slides were then covered with Fluorosafe reagent and immunofluorescent images were obtained on a Nikon TS100F fluorescent microscope.

### Transplantation of ADSC into Col6a1^−/−^Rag1^−/−^ mice under physiological and CTX-induced injury/regeneration conditions

All animal procedures were performed in accordance with the *Guide for the Care and Use of Laboratory Animals* (National Institutes of Health publication no. 86–23) and approved by the Institutional Animal Care and Use Committee of the Thomas Jefferson University. For all transplantation studies, ADSC (passage 2 to 4) were labeled with a red lipophilic tracer, DiOC18 (Molecular Probes, Grand Island, NY, USA). For therapeutic assessment of ADSC in physiological conditions, neonatal three- to five-day-old *Col6a1*^*−/−*^*Rag1*^*−/−*^ mice (n = 5/treatment/time point) were intramuscularly transplanted with 0.5 × 10^6^ DiOC18-ADSC in 50 μl PBS in the gastrocnemius muscle (GCM) of the left hindlimb. The right hindlimb was injected with PBS and served as a control. To induce muscle injury, *Col6a1*^*−/−*^*Rag1*^*−/−*^ mice (n = 5/treatment/time point) were injected with 10 μM cardiotoxin (CTX, Sigma) dissolved in PBS in the GCM of the left hindlimb two days after transplantation. The right GCMs were injected with PBS and used as a control. For analysis, transplanted mice were euthanized by CO_2_ inhalation at predetermined time points (one, two, three, four and six weeks) and muscle samples were collected. *In vivo* imaging (IVIS) (Lumina XR, Caliper LifeSciences, Hopkinton, MA, USA) was performed at indicated time points to determine the localization of the transplants. Transplantation was evaluated by histopathologic, indirect immunofluorescence and molecular analyses. For all histopathologic analyses, collected tissue samples were embedded into optimal cutting temperature (OCT) compound (VWR, Pittsburgh, PA, USA), frozen and cryosections taken at a thickness of 10 μm. For histological analysis, sections were stained with H & E using a standard protocol. All samples were evaluated for the presence of engrafted DiOC18-ADSC expressing cells using fluorescence microscopy. For indirect immunofluorescent analysis of collagen VI, cross-sections were stained with anti-α1(VI) collagen rabbit polyclonal antibodies. Immunocomplexes were detected with AlexaFluor^488^- or AlexaFluor^594^-labeled secondary antibodies (Invitrogen). For the analysis of co-localization of type VI collagen and lamin A/C-positive ADSC, muscle sections were stained with human specific monoclonal antibodies to lamin A/C (Millipore/Chemicon, Billerica, MA, USA) and AlexaFluor^488^-labeled anti-mouse immunoglobulin secondary antibodies (Invitrogen). For the analysis of co-localization of type VI and IV collagens, sections were stained with antibodies specific to type IV collagen (Millipore/Chemicon) and AlexaFluor^594^- or AlexaFluor^350^ labeled secondary antibodies (Invitrogen). To examine immune response, macrophages were detected with FITC-conjugated anti-mouse CD11b (BD Bioscience). Pax7-positive satellite cells were detected using anti-Pax7 (Developmental Studies Hybridome Bank, Iowa City, IA, USA). Images were taken and analyzed using AutoQuant imaging software (AutoQuant Imaging Inc, Troy, NY, USA). The relative number of the engrafted lamin A/C-positive ADSC on cryosections was determined by semi-quantitative immunofluorescence analysis using AutoQuant imaging software. The average number of collagen α1(VI) -positive fibers was calculated and compared between samples collected at different time points. Multiple adjacent sections were analyzed within 20 random, non-overlapping microscopic fields per sample. All morphometric comparisons are presented as percentages of untreated limb (baseline control) and analyzed for statistical significance using the Student’s t test, with *P* value less than 0.01 considered significant in all tests.

## Results

### Isolation and characterization of human adipose-derived stem cells

We developed a novel method for the isolation of ADSC from the neonatal skin discarded after newborn circumcision using negative immunodepletion with a cocktail of biotin-labeled lineage-specific antibodies and magnetic cell sorting. The original cultures of ADSC were produced as described in the Methods section. Routinely, approximately 1 × 10^6^ ADSC were produced from each skin sample after tissue digestion followed by negative selective depletion of mature hematopoietic cells, such as T cells, B cells, monocytes/macrophages, granulocytes, erythrocytes and their committed precursors. The ADSC appeared initially as adherent, spindle-shaped cells after three to four days of culture. Then, the cells grew rapidly, and could be easily expanded.

**Figure 1:**
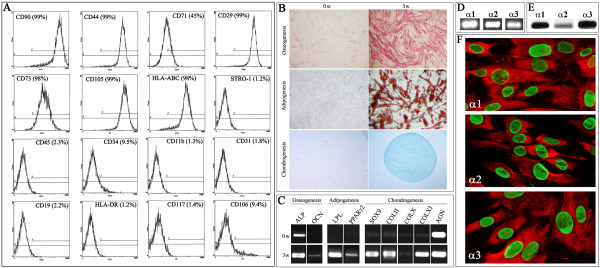
**Phenotypic analysis of primary human neonatal ADSC. (A)** Immunophenotypic analysis was performed using fluorescein-conjugated antibodies. For each sample, 10,000 events were read and the percent of positive cells expressing the respective surface marker is listed in the box. **(B)** Multilineage differentiation capacity of neonatal ADSC. Cells treated with osteogenic supplements showed an increased number of alkaline phosphatase-positive cells. Treatment of cells with adipogenic supplements resulted in the formation of adipocytic cells containing intracellular lipid droplets as detected by oil red staining. ADSC cultured in micromass showed a chondrogenic phenotype as detected by Alcian blue staining. **(C)** RT-PCR analysis of lineage-specific genes. Alkaline phosphatase (ALP) and osteocalcin (OCN) were used to indicate commitment to the osteogenic lineage. Lipoprotein lipase (LPL) and peroxisome proliferator-activated receptor gamma transcript variant 2 (PPARγ2) were used to indicate cells of an adipogenic lineage. SRY (sex determining region Y)-box 9 (SOX9), collagen type II (COLII), collagen type X (COLX), collagen type XI (COLXI), and aggrecan (AGN) were used to show commitment to the chondrogenic lineage. Top row shows the basal expression of these genes in control ADSC at 0 weeks. Bottom row shows expression of genes after three weeks of culturing in inductive media. **(D)** RT-PCR analysis for the expression of *COL6A1*, *COL6A2* and *COL6A3* genes in cultured ADSC using gene-specific primers. **(E)** Western blot analysis of individual (α1, α2, α3) collagen VI chains in ADSC using chain-specific antibodies. **(F)** Immunofluorescence analysis of individual (α1, α2, α3) chains of collagen VI was performed using chain-specific antibodies (red) and co-stained with human lamin A/C (green). ADSC, adipose-derived stem cells.

To determine the antigen expression profile of the established ADSC, immuno-phenotypic analysis (FACS) using fluorescently labeled antibodies against a panel of cell surface markers was performed (Figure 1A). The analysis showed that the cells do not express endothelial markers (CD31-PECAM1), hematopoietic markers (CD34, CD45 and CD117-c-kit) or the marker for macrophages (CD11b). However, the cells expressed high levels of CD44, CD105 (SH2), adhesion markers (CD29-integrin β1 and CD90-Thy-1) and mesenchymal stem cell marker CD73, consistent with the published profile from adult adipose tissues. Also, the ADSC were negative for HLA-class II (HLA-DR), but positive for HLA-class I (HLA-ABC).

To verify the ability of the generated ADSC cultures to differentiate into multiple cell types, the cells were analyzed for osteogenic, adipogenic and chondrogenic potentials *in vitro*. To induce osteogenic differentiation, the cells were maintained in differentiation basal osteogenic medium for up to five weeks. Cell cultures were morphologically examined and ALP activity and ECM calcification were assessed. As expected, the cells underwent a dramatic change in cellular morphology from spindle-shaped to cuboidal. In addition, histochemical analysis of the cells for ALP activity showed a high and uniform staining (Figure 1B). When the cultures were examined for their ability to elaborate a mineralized ECM, a substantial calcium deposition was detected, while no calcium deposition was seen in control ADSC cultures (data not shown). To verify these data, mRNA expression of the osteogenic markers, such as *ALP* and osteocalcin (*OCN*), was also investigated. As expected, cells under differentiation conditions showed substantial up-regulation of the indicated osteogenic marker genes (Figure 1C), although a low-level *ALP* expression could be detected in control non-differentiated cultures.

To induce adipocytic differentiation, cells were maintained in adipogenic induction medium for five weeks. For histochemical examination, the adipogenic culture was stained for the presence of intracellular lipid droplets as an indicator of intracellular lipid accumulation. Oil Red-O staining of five-week cultures for intracellular lipid vacuoles did not show any cells resembling adipocytes in control cultures, whereas upon treatment with adipogenic inductive medium, cells with a typical adipocyte phenotype containing intracellular lipid droplets appeared (Figure 1B). Moreover, RT-PCR analysis showed that adipocyte-related transcripts, such as lipoprotein lipase (*LPL*) and peroxisome proliferator-activated receptor γ2 (*PPARγ2*), were induced in differentiated ADSC (Figure 1C).

Chondrogenic differentiation was performed using the micromass culture technique in which cellular condensation (a critical first event of chondrogenesis) is duplicated. Chondrogenic medium was gently overlaid so as not to detach the cell nodules and the cultures were maintained for up to five weeks. Under these conditions, the cells formed nodules associated with a well-organized ECM rich in sulfated proteoglycans as detected using Alcian-blue staining under acidic conditions (Figure 1B). Nodules were associated with an Alcian-blue-positive ECM, indicative of the presence of sulfated proteoglycans within the matrix, whereas no obvious staining was observed in undifferentiated control ADSC cultures. Next, cell aggregates were analyzed by RT-PCR using specific primers for chondrogenic-related genes. As expected, aggregated cultures showed a marked induction of mRNAs from chondrogenic genes, including *SOX9* (Figure 1C). In addition, expression levels of *COL2A1* and *COL11A1* genes were also significantly increased. Overall, our data indicate that the neonatal ADSC generated here represent a morphologically homogenous population with phenotypic and functional features of adult mesenchymal stem cells and possess the capacity to differentiate toward specific lineages when exposed to the appropriate inductive media.

### Differential expression of ECM and related genes in ADSC

To examine the expression profile of the established ADSC, we performed a whole human genome oligo microarray analysis with a special emphasis on ECM, adhesion molecules and related genes. The median standard deviation of identified genes across three replicates was about 0.04, indicating good intra-array reproducibility. Transcriptional activity was identified in all categories of genes expressed by ADSC, including collagens, ECM and basement membrane constituents, adhesion molecules involved in cell-cell and cell-matrix interactions, and proteases involved in degradation of the ECM and their inhibitors (Table 1 and Additional file 2: Table S2). High expression of several collagen genes was detected, including those coding for the α1 and α2 chains of collagen I, the α2 chain of collagen IV, the α1 and α2 chains of collagen V, the α1 and α2 chains of collagen VI, the *α*1 chain of collagen XII, and the α1 chain of collagen XVI. Transcripts for many other collagens were also detectable. Moreover, relatively high expression of several basement membrane constituents was identified, such as the *LAMB1*, *LAMB2* and *LAMC1*. In addition, other genes with significant roles in ECM integrity were identified, such as the decorin (*DCN*, affects the fate of collagen fibril formation), lumican (*LUM*, regulates collagen fibril assembly) and elastin (*ELN*, the major component of elastic fibers, provides strength and flexibility to connective tissue). Among cell-matrix adhesion molecules detected were different integrins (*ITGA5*, *ITGA7*, *ITGAV*, *ITGB1* and *ITGB5*) and the immunoglobulin superfamily genes (*ICAM1*, *ICAM2* and *ICAM3*). The obtained transcriptional signatures also showed that ADSC express different transmembrane molecules, with the highest expression of the cadherin 2 (*CDH2*) and to a lesser extent *CDH11*, *CDH13* and *CHD22*. Several genes participating in ECM remodeling, including matrix metallopeptidases (*MMP1* and *MMP3*) and metallopeptidase inhibitors (*TIMP1, TIMP2* and *TIMP3*), were highly expressed in ADSC. Interestingly, the expression analysis of genes coding for myogenic markers showed that ADSC were negative for the majority of them, including myogenin, Myf5, MRF4, MyoD, Pax7 and M-cadherin. However, the analysis revealed that ADSC express a relatively high level of desmin (*DES,* helps maintain the structure of sarcomeres, which are necessary for muscle contraction) and low expression of the myocyte-specific enhancer factor 2C (*MEF2C*, plays a role in myogenesis and, possibly, in maintaining the differentiated state of muscle cells) and dystrophin (*DMD*, acts as an anchor for muscle fibers and protects them from injury). Taken together, our analysis shows that neonatal ADSC express a plethora of different ECM, adhesion molecules and other ECM-related genes.

**Table 1:** Differential expression analysis in primary human neonatal ADSC

**Gene symbol**	**RefSeq number**	**Description**	**Signal**	**Fold difference gene/GAPDH**^**a**^
**Extracellular matrix proteins**				
Collagens and ECM structural constituents				
COL1A1	NM_000088	Homo sapiens collagen, type I, alpha 1	183526	2.06
COL1A2	NM_000089	Homo sapiens collagen, type I, alpha 2	142138	1.59
COL3A1	NM_000090	Homo sapiens collagen, type III, alpha 1	3108	0.03
COL4A1	NM_001845	Homo sapiens collagen, type IV, alpha 1	5622	0.06
COL4A2	NM_001846	Homo sapiens collagen, type IV, alpha 2	44164	0.49
COL5A1	NM_000093	Homo sapiens collagen, type V, alpha 1	32129	0.36
COL5A2	NM_000393	Homo sapiens collagen, type V, alpha 2	19919	0.22
COL6A1	NM_001848	Homo sapiens collagen, type VI, alpha 1	117011	1.31
COL6A2	NM_058174	Homo sapiens collagen, type VI, alpha 2	11593	0.13
COL6A3	NM_004369	Homo sapiens collagen, type VI, alpha 3	1112	0.01
COL7A1	NM_000094	Homo sapiens collagen, type VII, alpha 1	1136	0.01
COL12A1	NM_004370	Homo sapiens collagen, type XII, alpha 1	17353	0.19
COL16A1	NM_001856	Homo sapiens collagen, type XVI, alpha 1	17366	0.19
DCN	NM_001920	Homo sapiens decorin	22826	0.25
FN1	NM_054034	Homo sapiens fibronectin 1	6213	0.07
LUM	NM_002345	Homo sapiens lumican	7747	0.09
Basement membrane constituents				
LAMA2	NM_000426	Homo sapiens laminin, alpha 2	804	0.01
LAMB1	NM_002291	Homo sapiens laminin, beta 1	10371	0.11
LAMB2	NM_002292	Homo sapiens laminin, beta 2 (laminin S)	16172	0.18
LAMC1	NM_002293	Homo sapiens laminin, gamma 1 (formerly LAMB2)	10072	0.11
**Cell adhesion molecules**				
Cell-matrix adhesion				
ITGA5	NM_002205	Homo sapiens integrin, alpha 5 (fibronectin receptor, alpha polypeptide)	3324	0.04
ITGA6	NM_000210	Homo sapiens integrin, alpha 6	1168	0.01
ITGA7	NM_002206	Homo sapiens integrin, alpha 7	7553	0.08
ITGB1	NM_133376	Homo sapiens integrin, beta 1 (fibronectin receptor, beta polypeptide, antigen CD29 includes MDF2, MSK12)	6616	0.07
ITGB5	NM_002213	Homo sapiens integrin, beta 5	4324	0.05
**ECM proteases and protease inhibitors**				
MMP1	NM_002421	Homo sapiens matrix metallopeptidase 1 (interstitial collagenase)	4368	0.05
TIMP1	NM_003254	Homo sapiens TIMP metallopeptidase inhibitor 1	42809	0.48
TIMP2	NM_003255	Homo sapiens TIMP metallopeptidase inhibitor 2	83481	0.93
TIMP3	NM_000362	Homo sapiens TIMP metallopeptidase inhibitor 3	36463	0.41
**Non-muscle and muscle markers**				
DES	NM_001927	Homo sapiens desmin	8119	0.09
MYO1C	NM_033375	Homo sapiens myosin IC	33956	0.38
MYLK	NM_053025	Homo sapiens myosin light chain kinase	12346	0.14
MYLK	NM_053025	Homo sapiens myosin light chain kinase	18846	0.21
MYH9	NM_002473	Homo sapiens myosin, heavy chain 9, non-muscle	15884	0.18
MYL6	NM_079423	Homo sapiens myosin, light chain 6, alkali, smooth muscle and non-muscle	51777	0.58

^a^The fold difference represents the ratio of intensity of each gene hybridized with the RNA isolated from hADSC normalized to the intensity of GAPDH gene. Each array was processed in an identical manner and the number represents an average of triplicate experiments from three independent cell isolates. Each gene is demarcated by the Genbank accession number, the description of the gene and the common name. Genes are grouped by their distinct functional categories. ADSC, adipose-derived stem cells; ECM, extracellular matrix.

Our gene expression analysis demonstrated that out of all collagen types detected in ADSC, collagen type VI (*COL6A1*, in particular) is the second most expressed gene after type I collagen (*COL1A1* and *COL1A2*). To further verify these data, expression of all three individual chains of collagen VI in established ADSC was confirmed by RT-PCR, western blot and immunofluorescence. The presence of *COL6A1*, *COL6A2* and *COL6A3* transcripts and proteins was detected using chain-specific primers and antibodies (Figure 1D and E), respectively. These results were further confirmed by indirect immunofluorescence analysis (Figure 1F). Overall, our data demonstrate that neonatal ADSC produce a significant level of collagen type VI and are readily available for therapeutic protein replacement in COL6 CMD.

### Intramuscular transplantation of ADSC into Col6a1^−/−^Rag1^−/−^ mice under physiological conditions

**Figure 2:**
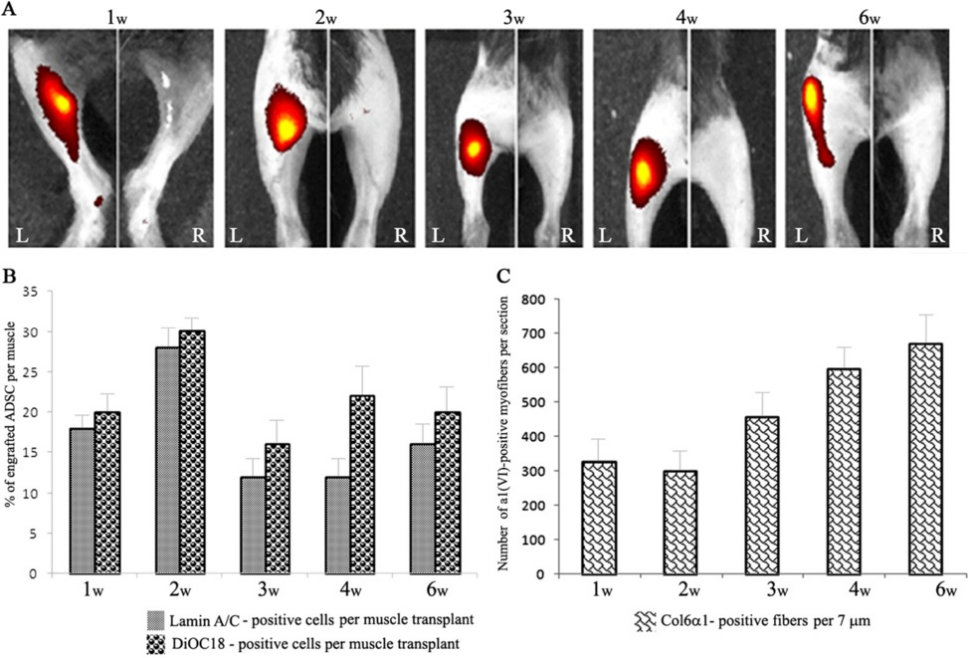
**Intramuscular transplantation of human neonatal ADSC into *Col6a1***^***−/−***^***Rag1***^***−/−***^**mice. (A)** Representative *in vivo* images showing the hindlimbs that received a single intramuscular injection of ADSC. Cells were cultured in the presence of a red fluorescent lipophilic dye (DiOC18), and 0.5 × 10^6^ DiOC18-ADSC were injected into the left GCM. The right GCM served as the control. IVIS imaging was performed at one, two, three, four and six weeks post-transplantation. **(B)** Morphometric analysis of DiOC18-ADSC engraftment into the GCM was performed at one, two, three, four and six weeks post-transplantation. The percentage of engrafted cells was determined by staining of transplanted GCM with lamin A/C antibody as a marker for donor cells and by counting lamin A/C positive-cells on at least fifty 7 μm sections. As an additional approach, the total number of DiOC18-positive ADSC was calculated using FACS analysis of a single-cell suspension of the GCM containing ADSC transplant. The vertical axis shows the percentage (%) of engrafted ADSC per injected muscle. The horizontal axis shows the time point after transplantation in weeks (w). **(C)** Morphometric analysis of α1(VI)-positive myofibers in the GCM transplanted with ADSC. The number of α1(VI)-positive myofibers was determined by counting a minimum of fifty 7 μm sections per animal. The vertical axis shows the number of α1(VI)-positive myofibers per section. The horizontal axis shows the time point after transplantation in weeks (w). In all studies, at least five animals were analyzed per time point. Error bars represent means ± SEM. ADSC, adipose-derived stem cells; FACS, fluorescence-activated cell sorting; GCM, gastrocnemius muscle; SEM, standard error of the mean.

**Figure 3:**
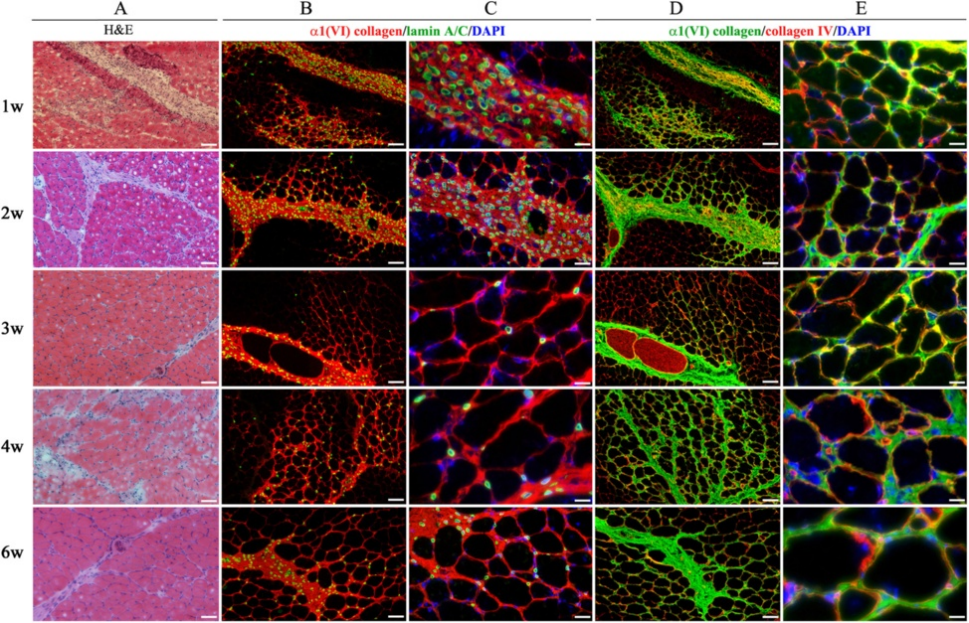
**Histochemical and indirect immunofluorescence analyses of GCM biopsies after transplantation of human neonatal ADSC into *Col6a1***^***−/−***^***Rag1***^***−/−***^**mice.** Muscle biopsies were collected at one, two, three, four, and six weeks after transplantation under homeostatic conditions. Time points are indicated to the left of the panels. **(A)** Hematoxylin and eosin staining (H & E) of the ADSC-treated GCM. **(B, C)** Indirect immunofluorescence detection of ADSC-derived collagen VI in the transplanted GCM. Donor cells were detected with anti-human lamin A/C antibodies (AlexaFluor^488^, green) and collagen VI-positive myofibers were detected with anti-α1(VI)-collagen antibodies (AlexaFluor^594^, red). Images were taken from representative sections at low **(B)** and high **(C)** magnification, respectively. **(D, E)** Co-localization of the ADSC-donated α1(VI)-collagen (AlexaFluor^488^, green) and basement-membrane-associated type IV collagen (AlexaFluor^594^, red) in ADSC-treated muscles. Images were taken from representative sections at low **(D)** and high **(E)** magnification, respectively. Nuclei were stained with DAPI (blue). Scale bar, 100 μm (low magnification) and 25 μm (high magnification), respectively. ADSC, adipose-derived stem cells; DAPI, 4′,6-diamidino-2-phenyl indol; GCM, gastrocnemius muscle.

**Figure 4:**
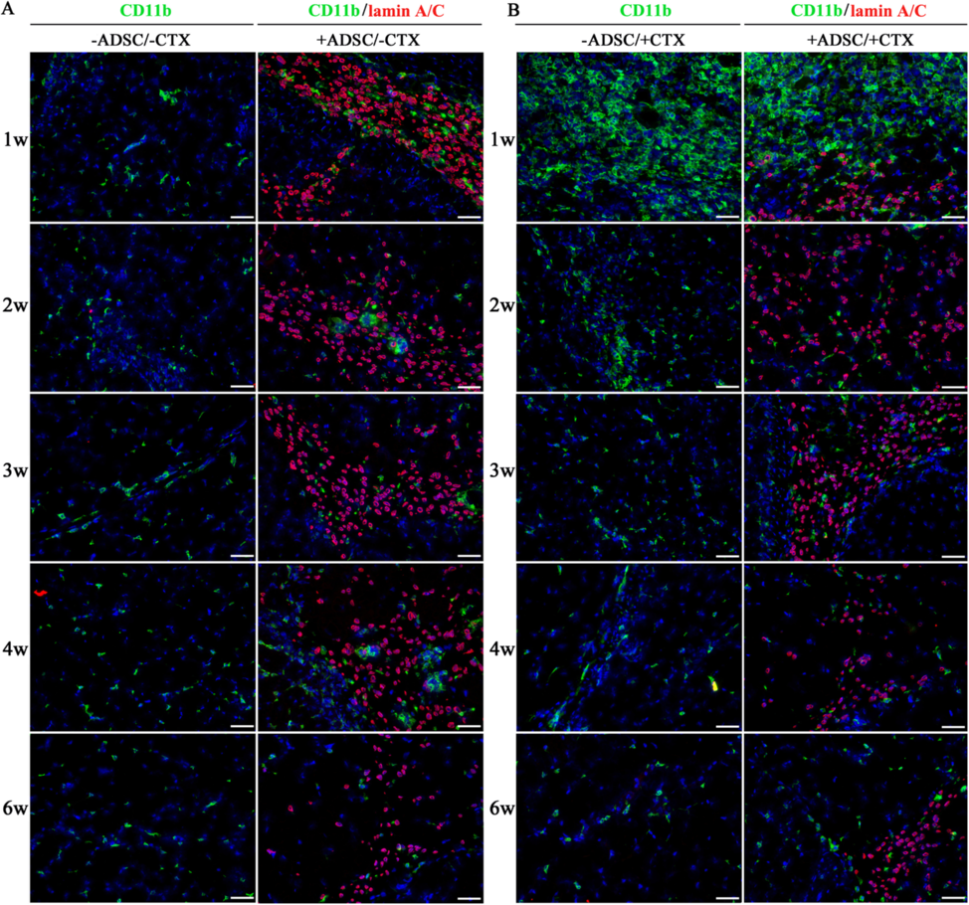
**Analysis of muscle tissue infiltration with CD11b-positive cells after ADSC transplantation. (A)** Indirect immunofluorescence assessment of the infiltrating CD11b-positive leukocytes (green) in untreated and ADSC-treated muscles of *Col6a1*^*−/−*^*Rag1*^*−/−*^ mice at different time points after transplantation. **(B)** Indirect immunofluorescence assessment of the infiltrating CD11b-positive leukocytes (green) in CTX-injured muscles with or without ADSC transplantation. In all cases, ADSC were detected with anti-human lamin A/C antibodies (red), whereas infiltrating CD11b-positive cells were detected with anti-CD11b antibodies (green). Time points are indicated to the left of the panels. Corresponding treatment is shown on top of the panels. Nuclei were stained with DAPI (blue). Scale bar, 100 μm. ADSC, adipose-derived stem cells; CTX, cardiotoxin; DAPI, 4′,6-diamidino-2-phenyl indol.

In order to assess the potential of neonatal ADSC in therapeutic application of COL6 CMD, fluorescently labeled ADSC (DiOC18-ADSC) were intramuscularly transplanted into the GCM of the left hindlimb of three- to five-day-old *Col6a1*^*−/−*^*Rag1*^*−/−*^ mice. The right limb was injected with PBS and served as internal control. Live imaging of both transplanted and control mouse limbs was performed every week for six weeks using the IVIS imaging system. To measure myofibers associated with immunoreactive α1(VI) collagen and estimate the engraftment level of donor cells in recipient muscle tissue, the cryosections with positive DiOC18-ADSC were co-immunostained with antibodies against α1(VI) collagen and lamin A/C as a marker for donor human cells. As shown in Figure 2A, the red fluorescent signals of DiOC18-labeled ADSC were detectable in all transplanted limbs throughout the study but not in the control hindlimbs. Based on morphometric analysis, the GCM receiving an ADSC transplant showed extensive engraftment as judged by the presence of lamin A/C-positive cells on muscle cryosections (Figure 2B). Moreover, we found that the level of engraftment did not change significantly and sustained at the level of 20% up to six weeks after transplantation. Analysis of the transplanted GCM of recipient limbs revealed a robust, time-dependent engraftment and distribution of collagen VI-positive myofibers (Figure 3). It is interesting to note that during the first week after transplantation the ADSC were preferentially found in the connective tissue of perimysium and the periphery of muscle fascicles with occasional cells in endomysial space. Furthermore, the presence of an excessive number of transplanted cells during initial engraftment stages resulted in substantial enlargement of perimysial space and significant production of collagen VI into adjacent tissue. Remarkably, in the next two to six weeks, prominent and continuous collagen VI staining was strongly associated with endomysial connective tissue and many surrounding individual myofibers, although, some transplanted cells continued to reside in the perimysium space (Figure 3B and C). The majority of the ADSC was able to migrate from primary perimysial residence to distal sites along endomysium, possibly in response to microenvironmental cues, and continued to donate therapeutic collagen VI protein to surrounding myofibers. In addition, the ADSC were positioned in close proximity to neighboring interstitial fibroblasts. Morphometric analysis showed a marked increase (two-fold) in α1(VI) collagen-positive myofibers at the sixth week as compared to the first week of transplantation (Figure 2C). It is well known that secreted collagen VI molecules form an extended microfilament network particularly abundant close to the cells, which has been suggested to play a role in anchoring the basement membrane of non-epithelial cells to the underlying connective tissue. Using double immunostaining with antibodies specific for type IV collagen, which is an integral component of the basement membrane, and α1(VI) collagen, we showed overlapping fluorescent signals of both proteins at the basement membrane of individual myofibers, suggesting functionality of the ADSC-secreted collagen VI protein (Figure 3D and E). *Col6a1*^*−/−*^ mice homozygous for the *Rag1* mutation are immunodeficient as they completely lack mature B and T lymphocytes but they display other fully functional inflammatory cells. To test whether transplantation with the ADSC resulted in any immune recognition, inflammatory cell infiltration was assessed by staining muscle tissue with CD11b, as a marker for macrophages, and lamin A/C, as a marker for donor cells. As seen in Figure 4A, a similar degree of infiltration by CD11b-positive cells was found in both untreated and ADSC-treated muscle. Overall, our data indicate that ADSC have engrafted, migrated and produced therapeutic collagen VI protein in muscle of *Col6a1*^*−/−*^*Rag1*^*−/−*^ mice.

### Intramuscular transplantation of ADSC into Col6a1^−/−^Rag1^−/−^ mice under injury/regeneration conditions

**Figure 5:**
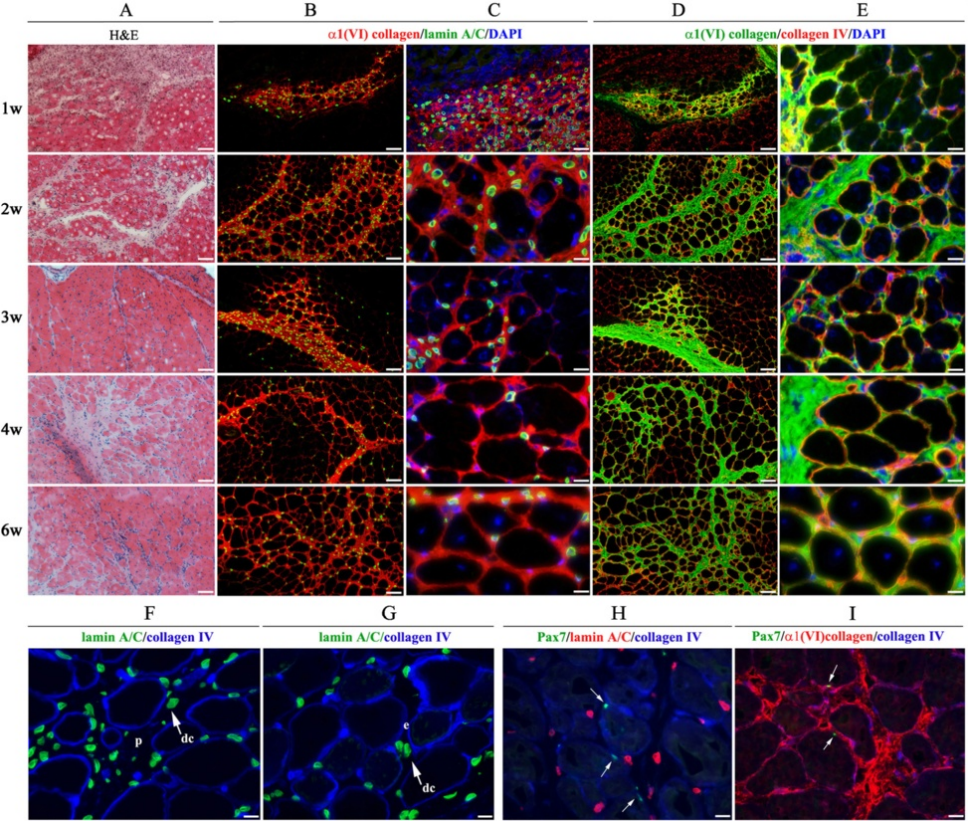
**Histochemical and indirect immunofluorescent analyses of GCM biopsies after ADSC transplantation and CTX injury.** Biopsies were collected at one, two, three, four and six weeks after ADSC transplantation (indicated to the left of the panels). **(A)** Hematoxylin and eosin staining (H & E) staining of ADSC-treated and CTX-injured GCM. **(B, C)** Indirect immunofluorescent detection of ADSC-derived collagen VI was performed using anti-lamin A/C (green) and anti-α1(VI) collagen (red) antibodies. Images were taken from representative sections at low **(B)** and high **(C)** magnification, respectively. Progressive, time-dependent spreading of transplanted ADSC within muscle tissue and donation of type VI collagen into perimysium and endomysium is apparent. **(D, E)** Indirect immunofluorescence detection of the α1(VI) collagen (green) and type IV collagen (red) co-localization at the basement membrane of the ADSC-treated, CTX-injured GCM. Images were taken from representative sections at low **(D)** and high **(E)** magnification, respectively. Nuclei were stained with DAPI (blue). Scale bar, 100 μm (low magnification) and 25 μm (high magnification), respectively. **(F, G)** Indirect immunofluorescence detection of lamin A/C (green) and type IV collagen (blue) in muscle biopsy after 30 days. Arrow points to donor cells (dc). P, perimysium; e, endomysium. **(H)** Indirect immunofluorescence detection of Pax7 (green), lamin A/C (red) and type IV collagen (blue) in muscle biopsy after 30 days. **(I)** Indirect immunofluorescence detection of Pax7 (green), α1(VI) collagen (red) and type IV collagen (blue) in muscle biopsy after 30 days. Arrows point to Pax7-positive (green) satellite cells. Scale bar, 100 μm. ADSC, adipose-derived stem cells; CTX, cardiotoxin; DAPI, 4′,6-diamidino-2-phenyl indol; GCM, gastrocnemius muscle.

**Figure 6:**
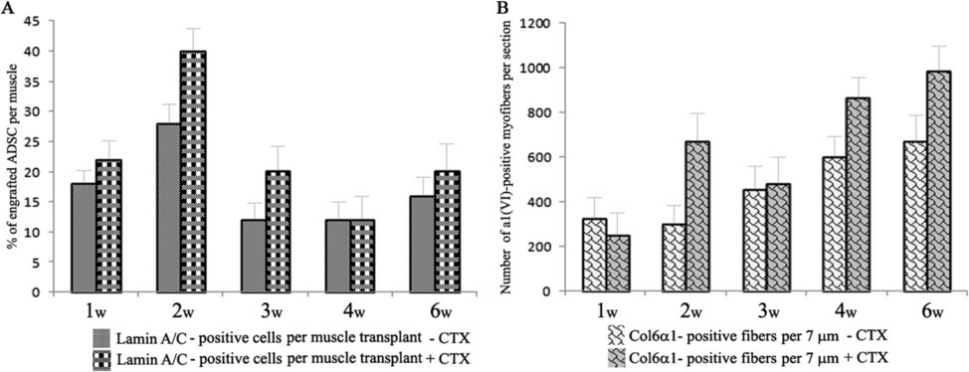
**Comparative morphometric analysis of ADSC engraftment and type VI collagen distribution in homeostatic and CTX-treated GCM, respectively.** Analysis was performed at one, two, three, four and six weeks post-transplantation and CTX injury. **(A)** Engraftment of ADSC into the GCM with or without CTX treatment. The vertical axis shows the percentage (%) of engrafted ADSC per injected muscle. The horizontal axis shows the time point after transplantation in weeks (w). **(B)** Morphometric analysis of α1(VI)-positive myofibers in the transplanted GCM with or without CTX treatment. The vertical axis shows the number of α1(VI)-positive myofibers per section. The horizontal axis shows the time point after transplantation in weeks (w). In all cases, ADSC were detected with anti-lamin A/C antibodies. The percentage of engrafted cells and the number of α1(VI)-positive myofibers were determined on at least fifty 7 μm cryosections, respectively, covering approximately 125 mm^3^ of muscle tissue. In all studies, at least five animals were analyzed per time point. Data is presented as average ± SD. ADSC, adipose-derived stem cells; CTX, cardiotoxin; GCM, gastrocnemius muscle; SD, standard deviation.

Since the muscle phenotype of the *Col6a1*^*−/−*^ mice is much milder than that of human patients, we used cardiotoxin (CTX) to injure the GCM muscle of the neonatal *Col6a1*^*−/−*^*Rag1*^*−/−*^ mice and thereby exacerbate the muscle abnormality. In order to enhance the regenerative response from donor stem cells, DiOC18-ADSC were injected into the left GCM two days prior to CTX injury and were traced at various time points after transplantation by systematic screening of histological sections. The right GCM was injected with CTX only as internal control. As expected, the direct injection of CTX into the GCM resulted in severe tissue injury in both control and ADSC transplanted muscle tissues. Histological analysis demonstrated global myofiber fragmentation and edema at one week after cell transplantation and five days after CTX treatment (Figure 5A). The CTX-injured muscle showed necrosis, disorganized muscle architecture and infiltration with mononucleated cells across the sectional area, mostly attributed to inflammatory cells (Figures 4B and 5A). Although the majority of transplanted cells were detected in perimysial space, small groups of the transplanted ADSC were readily found in close proximity to the injury site with already detectable collagen VI-positive myofibers. At two weeks post-ADSC injection, the CTX-treated muscles had numerous clusters of regenerating myofibers as judged by their small diameter, basophilic cytoplasm and centrally located nuclei. The level of engraftment did not change significantly and sustained at the level of 20% up to six weeks after transplantation (Figure 6A). Importantly, in CTX-treated muscles receiving ADSC transplant the number of collagen VI positive myofibers significantly increased (four-fold) in next three to six weeks (Figure 6B). Furthermore, the muscle morphology at three to six weeks post-ADSC injection/CTX injury was not different from that of the non-injured control muscles except for the presence of numerous myofibers with centrally positioned nuclei (Figures 5B-E), a known hallmark of recent muscle regeneration. Moreover, the number of CD11b infiltrating macrophages reduced dramatically, indicating the completion of the acute tissue damage phase (Figure 4B). The functionality of the ADSC-produced collagen VI was confirmed by double immunostaining with collagen α1 (VI) and IV antibodies, showing co-localization of both proteins at the basement membrane of individual myofibers (Figure 5D and E). It is well known that the muscle-specific stem cells, such as satellite cells, have a unique anatomical location and are positioned between the sarcolemma and the basal lamina of the muscle fibers. To see whether the transplanted ADSC can contribute to the satellite cell compartment and/or participate in formation of new myofibers, we examined the location of ADSC on the sections positively stained for donor engrafts in relation to the position of Pax7-positive satellite cells. In all examined sections of three and four week biopsies, human lamin A/C-positive cells were detected residing in the interstitial space of perimysium as well as endomysium surrounding individual muscle fibers (Figure 5F, G). Double-immunostaining with antibodies for human lamin Α/C and mouse Pax7 did not show any overlapping signals (Figure 5H), suggesting that donor cells had not entered the satellite cell niche or acquired muscle stem cell phenotype (Figure 5H). Moreover, regenerating and newly-formed collagen VI-positive myofibers did not show any lamin A/C-positive nuclei (Figure 5C), suggesting that the donor ADSC had not fused with mouse muscle fibers and had not directly participated in the formation of newly regenerating fibers. However, it was evident that ADSC-produced collagen VI was present at the basement membrane surrounding Pax7 cells (Figure 5I), suggesting that restored collagen VI expression may provide better mechanical properties to satellite cell niche and affect satellite cell activity. Overall, our data strongly suggest that collagen VI-donating ADSC were able to migrate intramuscularly in response to pro-migratory factors released in the course of CTX-induced injury and regeneration, and they continued to produce collagen VI protein in muscle of *Col6a1*^*−/−*^*Rag1*^*−/−*^ mice.

## Discussion

This study exploits the novel application of stem cell therapy specific for COL6 CMD. We tested the hypothesis that collagen VI secreted by ADSC in the ECM can ameliorate the genetic impairment of skeletal muscles in the COL6 CMD mouse model. We developed a novel procedure for isolation of ADSC from neonatal human skin discarded after newborn circumcision and demonstrated that established cultures represent a morphologically homogeneous population of cells with phenotypic and functional features of mesenchymal progenitors. Also, we conducted a comprehensive gene expression profile of ADSC with special emphasis on ECM and adhesion molecules. Furthermore, we showed for the first time that locally transplanted ADSC are capable of donating collagen VI protein into muscle of *Col6a1*^*−/−*^ mice under physiological and CTX-injury/regeneration conditions.

Adult stem cells represent a diverse group of stem cells, which are characterized by their ability to self-renew and their ability to differentiate along multiple lineage pathways. These unique features make these cells an attractive therapeutic option for transplantation and regeneration of damaged tissues. The most common type of adult stem cells is multipotent MSC, which can be isolated from several sources, including bone marrow, adipose tissue, dental pulp, placenta, umbilical cord and fallopian tube [23]. MSC isolated originally from bone marrow are defined as fibroblast-like cells with extensive proliferation capacity in culture and the ability to differentiate *in vitro* into various connective tissue cell types [28,29]. Recent advances in stem cell biology have shown that adult stem cells isolated from adipose tissue exhibit a number of properties suggesting the feasibility of their use as therapeutic cells [22]. The most important features of adipose tissue are its abundance in the body, relatively simple isolation procedures, expandability to relatively large quantities under minimal conditions and the ability to engraft after local or systemic reintroduction [22,23].

Here, we developed a novel procedure for the isolation of ADSC from neonatal human skin discarded after newborn circumcision and demonstrated that established cultures represent population of cells with features of mesenchymal progenitors. The developed isolation protocol proved to be a reliable approach to prepare a homogeneous fraction of human ADSC. Our antigenic cell surface profile is consistent with a previously published immunophenotypic analysis available for adult stem cells [23]. A hallmark characteristic of stem cells is their self-renewal potential and ability to differentiate into several cell lineages *in vitro* with the appropriate inductive media. The established ADSC cultures were able to differentiate toward adipogenic, osteogenic and chondrogenic lineages *in vitro* in the presence of lineage specific differentiation factors. Successful differentiation was confirmed by morphological changes demonstrated using lineage specific staining and gene expression analysis of genetic markers for the cell type of interest. Overall, these results are consistent with previously published works.

Multiple gene expression profiling studies have shown that adult stem cells, in particular MSC, can produce a vast array of proteins, including growth factors, cytokines, ECM and adhesion molecules [30]. However, no data are available regarding the expression of ECM and related genes by human neonatal ADSC. Our comprehensive gene expression analysis has shown that neonatal ADSC express a plethora of various ECM molecules, including many collagens and laminins, as well as adhesion molecules and other ECM-related genes. Of special interest to this study is collagen VI, ultimate cause of COL6 CMD. We show that ADSC produce a significant level of all three collagen VI mRNAs, in particular *COL6A1*. The use of ADSC for the treatment of COL6 CMD thus seems to be a logical choice since the cells are able to supply continuously the missing normal collagen VI protein.

Cell-based therapy has been explored for different types of muscular dystrophies, in particular, Duchenne muscular dystrophy [19,31]. The majority of these studies evaluated muscle derived progenitor or stem cells, such as myoblasts, side population cells, myogenic endothelial cells and mesoangioblasts. Several studies have tested stem cells derived from non-muscle tissues [32-34]. The transplanted cells were found to have limited ability to regenerate muscle fibers but were capable of reducing inflammation through trophic effects produced by transplanted cells [33,35]. Thus, even though a range of therapeutic approaches have been investigated for traditional muscular dystrophies, there is a need to develop treatment strategies that specifically target muscle ECM alterations. In the skeletal muscle, collagen VI is synthesized by muscle fibroblasts rather than muscle cells [8]. This is in stark contrast to most other muscular dystrophies in which the gene mutations usually involve intracellular or cell surface proteins produced by muscle cells. Therefore, the ability of stem cells to differentiate into muscle cells is not crucial for the therapeutic intervention of COL6 CMD. Moreover, the multi-lineage differentiation capacity of these cells is ideally suited for therapeutic intervention of COL6 CMD in which different connective tissue cell types need to be replenished. Also, apart from the cell regeneration capacity, an emerging therapeutic application of the stem cell therapy takes advantage of their ability to secrete cytokines and growth factors that are anti-apoptotic, pro-angiogenic, anti-inflammatory and anti-fibrotic [36]. Increased muscle cell apoptosis and interstitial fibrosis are typical pathological features of CMDs; therefore, stem cell transplantation likely will be beneficial even without cell differentiation and regeneration.

In this study, the therapeutic potential of neonatal ADSC was examined after local intramuscular injection into the GCM of *Col6a1*^*−/−*^*Rag1*^*−/−*^ mice, allowing human cell transplantation without immune rejection. Our study has shown for the first time that ADSC contributed robustly to the collagen VI-deficient GCM by providing efficient engraftment, wide-distribution of therapeutic cells in muscle tissue, continuous production of collagen VI protein by engrafted cells throughout the observation period and proper assembly of collagen VI microfibrils in the interstitial connective tissue of muscle, thus achieving restoration of the affected muscle basement membrane. One of the striking observations is the location of donor cells within the muscle tissue at initial stages of transplantation. During the first two weeks, the vast majority of ADSC was preferentially detected in perimysial space with very few cells in endomysium. Such unique locale could be explained by the fact that the muscle ECM, which is rich in connective tissue and adhesion proteins, may provide a proper environment for firm anchorage/attachment of ADSC. Once cells are acclimated to the perimysial environment, they start to migrate along endomysium, possibly in response to pro-migratory factors secreted by resident interstitial fibroblasts and muscle cells. Importantly, the number of α1(VI) collagen-positive myofibers was markedly increased (two-fold) at week six as compared to the first week of transplantation, suggesting the possibility of long-term commitment of ADSC in the muscle environment.

The capacity of neonatal ADSC to participate in the regeneration of skeletal muscle was further evaluated using the CTX-induced myonecrosis model with actively ongoing regeneration and remodeling of muscle tissue. Several important observations were made during these studies. It is believed that the depletion of the satellite cell pool with consequent irreversible muscle degeneration is responsible for terminal muscle failure in DMD, COL6 CMD and, possibly, other muscular dystrophies [37,38]. Restoration of the regeneration potential of muscle tissue by transplanted stem cells may provide long-term efficacy for muscle homeostasis. This notion is strongly supported by a recent observation that transplantation of wild-type fibroblasts into Col6a1 null mice was able to ameliorate regeneration and satellite cell homeostasis by restoring muscle mechanical properties and satellite cell activity [38]. However, these effects may be somehow limited by the fact that the grafted cells are differentiated fibroblasts. Our data clearly demonstrate that ADSC are able to colonize in muscle, sustaining their long-term maintenance and the continuous replenishment of collagen VI. However, our data demonstrated that ADSC do not directly participate in the repair of CTX-damaged muscle fibers. Immunofluorescent analysis did not show any ADSC entering the satellite niche, as judged by the position of lamin A/C-positive cells in newly regenerated myofibers. In addition, double-immunostaining of CTX-treated biopsies with lamin A/C (donor human cells) and Pax7 (a marker for both quiescent and activated satellite cells) antibodies did not show any overlapping signals. Taken together, these data suggest that ADSC may not possess cell-autonomous properties supporting myogenic differentiation *in vivo*. Also, it may suggest that CTX treatment alone does not induce or enhance production of supporting factors to provide a more conducive environment for ADSC to go through myogenic commitment. Nevertheless, CTX-treated muscles receiving the ADSC transplant showed a significant increase (four-fold) in the number of collagen VI-positive myofibers at the sixth week compared to the first week of transplantation and a two-fold increase compared to transplanted muscle without CTX-injury at the sixth week. It is important to note that ADSC engraftment was not dependent on ablative injury, as cells also contributed at high efficiency (approximately 20%) after transplantation into the GCM and sustained at this level throughout the study. However, it is interesting to note that after CTX-injury collagen VI-donating ADSC were able to migrate intramuscularly from their initial perimysial locale shortly after transplantation to the CTX-injected site, and eventually repopulated a significant area of the damaged tissue and adjacent myofibers. This directional migration from perimysium to endomysium could possibly be induced in response to pro-migratory factors released in the course of CTX-injury and regeneration, elicited by various mechanisms. As one explanation, it is likely that pro-inflammatory cytokines, chemokines and growth factors that typically result in homing of immune cells to a damaged site are released from muscle resident cells, stimulating directional migration of ADSC within muscle tissue. In fact, a prominent feature of CTX-injured muscle is a striking inflammatory infiltrate of immune cells, such as macrophages and neutrophils. Also, it is well known that CCR2 and CXCR2 are major regulators of induced macrophage and neutrophils trafficking *in vivo*[39,40]. In addition, the CXCR4-CXCL12 chemotactic axis was shown to play a significant role in regulating migration of both proliferating and terminally differentiated muscle cells [41]. Based on our assessment of chemokine receptors on ADSC, it is plausible that ADSC expressing those receptors can be recruited to the CTX-damaged site by the chemotactic mechanisms of inflammatory and satellite cells, respectively. Also, it is possible that abnormal signaling due to the deficient ECM leads to secretion of various chemokines, which may activate corresponding receptors on ADSC and facilitate migration toward the highest concentration of chemokine(s). Together, these observations suggest that mobilization of ADSC in CTX-damaged muscle may highly depend on the local inflammatory state rather than other factors. The ability of ADSC to donate the protein of interest to the injured muscle strongly supports the possibility of stem cell therapy for COL6 CMD, which exhibits substantially more severe myopathology than the mouse model.

## Conclusions

In summary, our data provided proof-of-concept that ADSC can serve as a ‘cell factory’ to produce ECM molecules, such as collagen VI, which can potentially correct the protein deficiency of COL6 CMD patients. Our data also suggest that a systemic administration of stem cells can be beneficial to counteract the disease phenotype in the different muscles. A significant obstacle in designing stem cell-based therapy for COL6 CMD and other muscular dystrophies is the necessity to reach the entire body musculature, a problem that cannot be easily overcome unless systemic transplantation protocols are proved to be effective. Further investigations are required to address these important issues. Our results provide evidence supporting the suitability of ADSC as a prospective therapy for COL6 CMD. If successful, the therapeutic capabilities of stem cells could be extended to other ECM-related muscular dystrophies that are in need of more effective treatment options.

## Abbreviations

ADSC: adipose-derived stem cells; ALP: alkaline phosphatase; BSA: bovine serum albumin; CMD: congenital muscular dystrophy; COL6 CMD: collagen VI congenital muscular dystrophy; CTX: cardiotoxin; DAPI: 4′,6-diamidino-2-phenyl indol; (D)MEM/F12: (Dulbecco’s) modified Eagle’s medium; ECM: extracellular matrix; EDTA: ethylenediaminetetraacetic acid; FACS: fluorescence-activated cell sorting; FBS: fetal bovine serum; FITC: fluorescein isothiocyanate; GCM: gastrocnemius muscle; H & E: hematoxylin and eosin; MSC: mesenchymal stem cells; PBS: phosphate-buffered saline; RT-PCR: reverse-transcriptase polymerase chain reaction; UCMD: Ullrich congenital muscular dystrophy.

## Competing interests

The authors declare that they have no competing interests.

## Authors’ contributions

VA participated in the design of experiments, carried out the molecular analysis of cells, cell transplantation into animals, interpretation and analysis of *in vitro* and *in vivo* data, and helped to draft the manuscript. MA and AD participated in all experiments involving animals, including colony maintenance, genotyping, collection of biopsies, histological and immunofluorescence analyses. PB conceived of the study, participated in its design and helped to draft the manuscript. OI and MLC have been involved in all aspects of the study, including experimental design, characterization of cells, gene expression study, transplantation studies, analysis and interpretation of data and manuscript writing. All authors read and approved the final manuscript.

## Supplementary Material

Additional file 1: Table S1 Microsoft Word. A table presenting the sequences of primers used in the study. Click here for file

Additional file 2: Table S2 Microsoft Word. A table presenting the differential expression analysis in primary human neonatal ADSC. Click here for file
