# Supplementary material for: Human adipose-derived stem cell transplantation as a potential therapy for collagen VI-related congenital muscular dystrophy
Source: Stem Cell Res Ther. 2014 Feb 12;5(1):21. doi: 10.1186/scrt411 (PMC4054951; doi:10.1186/scrt411)
Supplement: Additional file 1: Table S1 — Microsoft Word. A table presenting the sequences of primers used in the study. [file scrt411-S1.doc]

Table 1S: Primers used in the study.

| **Primer Name** | **Sequence** | **Amplicon Size (bp)** | **Tm (°C)** |
| --- | --- | --- | --- |
| ALP Forward | 5’-TGGAGCTTCAGAAGCTCAACACCA-3’ | 453 | 56 |
| ALP Reverse | 5’-ATCTCGTTGTCTGAGTACCAGTCC-3’ |  |  |
| BGLAP/OCN Forward | 5’-ATGAGAGCCCTCACACTCCTC-3’ | 297 | 60 |
| BGLAP/OCN Reverse | 5’-GCCGTAGAAGCGCCGATAGGC-3’ |  |  |
| LPL Forward | 5’-GAGATTTCTCTGTATGGCACC-3’ | 276 | 56 |
| LPL Reverse | 5’-CTGCAAATGAGACACTTTCTC-3’ |  |  |
| PPARγ2 Forward | 5’-GCTGTTATGGGTGAAACTCTG-3’ | 352 | 56 |
| PPARγ2 Reverse | 5’-ATAAGGTGGAGATGCAGGCTC-3’ |  |  |
| SOX9 Forward | 5’-ATCTGAAGAAGGAGAGCGAG-3’ | 264 | 56 |
| SOX9 Reverse | 5’-TCAGAAGTCTCCAGAGCTTG-3’ |  |  |
| COLII Forward | 5’-CCGCGGTGAGCCATGATTCG-3’ | 377(A)/171(B) | 60 |
| COL2II Reverse | 5’-CAGGCCCAGGAGGTCCTTTGGG-3’ |  |  |
| COLX Forward | 5’-GCCCAAGAGGTGCCCCTGGAATAC-3’ | 703 | 60 |
| COLX Reverse | 5’-CCTGAGAAAGAGGAGTGGACATAC-3’ |  |  |
| COLXI Forward | 5’-GGAAAGGACGAAGTTGGTCTGC-3’ | 590 | 60 |
| COLXI Reverse | 5’-TTCTCCACGCTGATTGCTACCC-3’ |  |  |
| COL6A1 Forward  COL6A1 Reverse | 5'-TCAGAATAGTGATGTGTTCGACGTT-3'  3'-AGCAACATGGATATGGTTCAGAAA-5' | 101 | 60 |
| COL6A2 Forward  COL6A2 Reverse | 5'-GACTGTGAGAAGCGCTGTGG-3'  3'-TCTGTTTGGCAGGGAAGGTC-5' | 647 | 60 |
| COL6A3 Forward  COL6A3 Reverse | 5'-GCAGTGCCATTGAATACACC-3'  3'-CCAGGACCACGAAGAAGTAG-5' | 170 | 60 |
| ACAN/AGN Forward | 5’-TGAGGAGGGCTGGAACAAGTACC-3’ | 350 | 56 |
| ACAN/AGN Reverse | 5’-GGAGGTGGTAATTGCAGGGAACA-3’ |  |  |
| GAPDH Forward | 5'-AACGTGTCAGTGGTGGACCT-3' | 250 | 56 |
| GAPDH Reverse | 5'-TGCTGTAGCCAAATTCGTTG-3' |  |  |
