# Supplementary material for: Human adipose-derived stem cell transplantation as a potential therapy for collagen VI-related congenital muscular dystrophy
Source: Stem Cell Res Ther. 2014 Feb 12;5(1):21. doi: 10.1186/scrt411 (PMC4054951; doi:10.1186/scrt411)
Supplement: Additional file 2: Table S2 — Microsoft Word. A table presenting the differential expression analysis in primary human neonatal ADSC. [file scrt411-S2.doc]

Table S2. Differential expression analysis in primary human neonatal ADSC.

| **Gene Symbol** | **RefSeq Number** | **Description** | **Signal** | **Fold difference**  **Gene/GAPDH^a^** |
| --- | --- | --- | --- | --- |
| **Extracellular Matrix Proteins** | | | | |
| *Collagens and ECM Structural Constituents* | | | | |
| CCBE1 | NM_133459 | Homo sapiens collagen and calcium binding EGF domains 1 | 394 | 0.004414814 |
| COL1A1 | NM_000088 | Homo sapiens collagen, type I, alpha 1 | 183526 | 2.056978402 |
| COL1A2 | NM_000089 | Homo sapiens collagen, type I, alpha 2 | 142138 | 1.593095235 |
| COL3A1 | NM_000090 | Homo sapiens collagen, type III, alpha 1 | 3108 | 0.034835853 |
| COL4A1 | NM_001845 | Homo sapiens collagen, type IV, alpha 1 | 5622 | 0.063016963 |
| COL4A2 | NM_001846 | Homo sapiens collagen, type IV, alpha 2 | 44164 | 0.495000392 |
| COL4A3BP | NM_001130105 | Homo sapiens collagen, type IV, alpha 3 binding protein | 1050 | 0.011763125 |
| COL4A4 | NM_000092 | Homo sapiens collagen, type IV, alpha 4 | 242 | 0.002709877 |
| COL4A5 | NM_033380 | Homo sapiens collagen, type IV, alpha 5 | 514 | 0.005755374 |
| COL9A3 | NM_001853 | Homo sapiens collagen, type IX, alpha 3 | 262 | 0.002932043 |
| COL5A1 | NM_000093 | Homo sapiens collagen, type V, alpha 1 | 32129 | 0.360110008 |
| COL5A2 | NM_000393 | Homo sapiens collagen, type V, alpha 2 | 19919 | 0.223253561 |
| COL5A3 | NM_015719 | Homo sapiens collagen, type V, alpha 3 | 315 | 0.003526779 |
| COL6A1 | NM_001848 | Homo sapiens collagen, type VI, alpha 1 | 117011 | 1.311470282 |
| COL6A2 | NM_058174 | Homo sapiens collagen, type VI, alpha 2 | 11593 | 0.129936181 |
| COL6A3 | NM_004369 | Homo sapiens collagen, type VI, alpha 3 | 1112 | 0.01246613 |
| COL7A1 | NM_000094 | Homo sapiens collagen, type VII, alpha 1 | 1136 | 0.012735145 |
| COL8A1 | NM_001850 | Homo sapiens collagen, type VIII, alpha 1 | 1708 | 0.019147528 |
| COL8A2 | NM_005202 | Homo sapiens collagen, type VIII, alpha 2 | 391 | 0.004387553 |
| COL11A2 | NM_001163771 | Homo sapiens collagen, type XI, alpha 2 | 255 | 0.002854479 |
| COL12A1 | NM_004370 | Homo sapiens collagen, type XII, alpha 1 | 17353 | 0.194492463 |
| COL13A1 | NM_080801 | Homo sapiens collagen, type XIII, alpha 1 | 845 | 0.009467612 |
| COL15A1 | NM_001855 | Homo sapiens collagen, type XV, alpha 1 | 844 | 0.009462863 |
| COL16A1 | NM_001856 | Homo sapiens collagen, type XVI, alpha 1 | 17366 | 0.194643335 |
| COL18A1 | NM_030582 | Homo sapiens collagen, type XVIII, alpha 1 | 6260 | 0.070164434 |
| COL23A1 | CU692082 | collagen, type XXIII, alpha 1 | 428 | 0.004801349 |
| COL27A1 | AK021957 | collagen, type XXVII, alpha 1 | 926 | 0.010381375 |
| CTHRC1 | NM_138455 | Homo sapiens collagen triple helix repeat containing 1 | 2350 | 0.026338103 |
| DCN | NM_001920 | Homo sapiens decorin | 22826 | 0.255839208 |
| FN1 | NM_054034 | Homo sapiens fibronectin 1 | 6213 | 0.069640208 |
| ELN | NM_000501 | Homo sapiens elastin | 980 | 0.010983735 |
| LUM | NM_002345 | Homo sapiens lumican | 7747 | 0.086823834 |
| *Basement Membrane Constituents* | | | | |
| LAMA1 | NM_005559 | Homo sapiens laminin, alpha 1 | 201 | 0.002252344 |
| LAMA2 | NM_000426 | Homo sapiens laminin, alpha 2 | 804 | 0.009012385 |
| LAMA3 | NM_198129 | Homo sapiens laminin, alpha 3 | 287 | 0.003219581 |
| LAMA4 | NM_001105209 | Homo sapiens laminin, alpha 4 | 1949 | 0.021842494 |
| LAMB1 | NM_002291 | Homo sapiens laminin, beta 1 | 10371 | 0.116242499 |
| LAMB2 | NM_002292 | Homo sapiens laminin, beta 2 (laminin S) | 16172 | 0.181253012 |
| LAMB3 | NM_001017402 | Homo sapiens laminin, beta 3 | 323 | 0.003624462 |
| LAMC1 | NM_002293 | Homo sapiens laminin, gamma 1 (formerly LAMB2) | 10072 | 0.112888434 |
| LAMC2 | NM_005562 | Homo sapiens laminin, gamma 2 | 882 | 0.009887596 |
| **Cell Adhesion Molecules** | | | | |
| *Cell-matrix adhesion* | | | | |
| ICAM1 | NM_000201 | Homo sapiens intercellular adhesion molecule 1 | 337 | 0.0037798 |
| ICAM2 | NM_000873 | Homo sapiens intercellular adhesion molecule 2 | 237 | 0.0026542 |
| ICAM3 | NM_002162 | Homo sapiens intercellular adhesion molecule 3 | 783 | 0.0087805 |
| ITFG1 | NM_030790 | Homo sapiens integrin alpha FG-GAP repeat containing 1 | 1575 | 0.017655264 |
| ITFG2 | NM_018463 | Homo sapiens integrin alpha FG-GAP repeat containing 2 | 474 | 0.005317616 |
| ITFG3 | NM_032039 | Homo sapiens integrin alpha FG-GAP repeat containing 3 | 5782 | 0.064806727 |
| ITGB1BP2 | NM_012278 | Homo sapiens integrin beta 1 binding protein (melusin) 2 | 136 | 0.001524497 |
| ITGB1BP1 | NM_022334 | Homo sapiens integrin beta 1 binding protein 1 | 7135 | 0.079964757 |
| ITGB3BP | NM_014288 | Homo sapiens integrin beta 3 binding protein (beta3-endonexin) | 514 | 0.005756553 |
| ITGA1 | NM_181501 | Homo sapiens integrin, alpha 1 | 422 | 0.004728717 |
| ITGA10 | NM_003637 | Homo sapiens integrin, alpha 10 | 166 | 0.001858315 |
| ITGA11 | NM_001004439 | Homo sapiens integrin, alpha 11 | 1280 | 0.014351519 |
| ITGA3 | NM_002204 | Homo sapiens integrin, alpha 3 (antigen CD49C, alpha 3 subunit of VLA-3 receptor) | 662 | 0.00741463 |
| ITGA4 | NM_000885 | Homo sapiens integrin, alpha 4 (antigen CD49D, alpha 4 subunit of VLA-4 receptor) | 822 | 0.009209932 |
| ITGA5 | NM_002205 | Homo sapiens integrin, alpha 5 (fibronectin receptor, alpha polypeptide) | 3324 | 0.037257578 |
| ITGA6 | NM_000210 | Homo sapiens integrin, alpha 6 | 1168 | 0.01309021 |
| ITGA7 | NM_002206 | Homo sapiens integrin, alpha 7 | 7553 | 0.084659511 |
| ITGAE | NM_002208 | Homo sapiens integrin, alpha E (antigen CD103, human mucosal lymphocyte antigen 1; alpha polypeptide) | 1155 | 0.012941193 |
| ITGAV | NM_002210 | Homo sapiens integrin, alpha V (vitronectin receptor, alpha polypeptide, antigen CD51) | 4923 | 0.055180059 |
| ITGB1 | NM_133376 | Homo sapiens integrin, beta 1 (fibronectin receptor, beta polypeptide, antigen CD29 includes MDF2, MSK12) | 6616 | 0.074152767 |
| ITGB3 | NM_000212 | Homo sapiens integrin, beta 3 (platelet glycoprotein IIIa, antigen CD61) | 321 | 0.003601434 |
| ITGB5 | NM_002213 | Homo sapiens integrin, beta 5 | 4324 | 0.04846312 |
| ITGB7 | NM_000889 | Homo sapiens integrin, beta 7 | 164 | 0.001837495 |
| ITGBL1 | NM_004791 | Homo sapiens integrin, beta-like 1 (with EGF-like repeat domains) | 3923 | 0.043965214 |
| ILK | NM_001014795 | Homo sapiens integrin-linked kinase | 3432 | 0.03846931 |
| ILKAP | NM_030768 | Homo sapiens integrin-linked kinase-associated serine/threonine phosphatase | 167 | 0.001866682 |
| *Transmembrane Molecules* | | | | |
| CDH11 | NM_001797 | Homo sapiens cadherin 11, type 2, OB-cadherin (osteoblast) | 6051 | 0.06782098 |
| CDH13 | NM_001257 | Homo sapiens cadherin 13, H-cadherin (heart) | 1100 | 0.012331657 |
| CDH2 | NM_001792 | Homo sapiens cadherin 2, type 1, N-cadherin (neuronal) | 18967 | 0.212586544 |
| CDH22 | NM_021248 | Homo sapiens cadherin 22, type 2 | 3640 | 0.040795754 |
| CDH24 | NM_022478 | Homo sapiens cadherin 24, type 2 | 111 | 0.001240673 |
| CDH3 | NM_001793 | Homo sapiens cadherin 3, type 1, P-cadherin (placental) | 129 | 0.001447215 |
| CDH4 | NM_001794 | Homo sapiens cadherin 4, type 1, R-cadherin (retinal) | 302 | 0.003389409 |
| CDH6 | NM_004932 | Homo sapiens cadherin 6, type 2, K-cadherin (fetal kidney) | 331 | 0.003713368 |
| **ECM Proteases and Protease Inhibitors** | | | | |
| MMP1 | NM_002421 | Homo sapiens matrix metallopeptidase 1 (interstitial collagenase) | 4368 | 0.048957768 |
| MMP14 | NM_004995 | Homo sapiens matrix metallopeptidase 14 (membrane-inserted) | 1206 | 0.013516856 |
| MMP15 | NM_002428 | Homo sapiens matrix metallopeptidase 15 (membrane-inserted) | 108 | 0.001208424 |
| MMP16 | NM_005941 | Homo sapiens matrix metallopeptidase 16 (membrane-inserted) | 163 | 0.001824977 |
| MMP17 | NM_016155 | Homo sapiens matrix metallopeptidase 17 (membrane-inserted) | 1492 | 0.016718827 |
| MMP19 | NM_002429 | Homo sapiens matrix metallopeptidase 19 | 122 | 0.001362087 |
| MMP19 | NM_002429 | Homo sapiens matrix metallopeptidase 19 | 935 | 0.010478734 |
| MMP2 | NM_004530 | Homo sapiens matrix metallopeptidase 2 | 3034 | 0.034007173 |
| MMP23B | NM_006983 | Homo sapiens matrix metallopeptidase 23B | 336 | 0.003770065 |
| MMP24 | NM_006690 | Homo sapiens matrix metallopeptidase 24 (membrane-inserted) | 185 | 0.002071238 |
| MMP28 | NM_001032278 | Homo sapiens matrix metallopeptidase 28 | 174 | 0.001948567 |
| MMP3 | NM_002422 | Homo sapiens matrix metallopeptidase 3 (stromelysin 1, progelatinase) | 22901 | 0.256677542 |
| TIMP1 | NM_003254 | Homo sapiens TIMP metallopeptidase inhibitor 1 | 42809 | 0.479803 |
| TIMP2 | NM_003255 | Homo sapiens TIMP metallopeptidase inhibitor 2 | 83481 | 0.935667 |
| TIMP3 | NM_000362 | Homo sapiens TIMP metallopeptidase inhibitor 3 | 36463 | 0.408677 |
| TIMP4 | NM_003256 | Homo sapiens TIMP metallopeptidase inhibitor 4 | 198 | 0.002219 |
| **Chemokines and Receptors** | | | | |
| CCL16 | NM_004590 | Homo sapiens chemokine (C-C motif) ligand 16 | 637 | 0.007136 |
| CCL2 | NM_002982 | Homo sapiens chemokine (C-C motif) ligand 2 | 5039 | 0.056475 |
| CCL24 | NM_002991 | Homo sapiens chemokine (C-C motif) ligand 24 | 2590 | 0.029031 |
| CCR10 | NM_016602 | Homo sapiens chemokine (C-C motif) receptor 10 | 731 | 0.008198 |
| CCR6 | NM_031409 | Homo sapiens chemokine (C-C motif) receptor 6 | 157 | 0.001764 |
| CCRL1 | NM_178445 | Homo sapiens chemokine (C-C motif) receptor-like 1 | 517 | 0.005791 |
| CXCL1 | NM_001511 | Homo sapiens chemokine (C-X-C motif) ligand 1 (melanoma growth stimulating activity, alpha) | 1598 | 0.017905 |
| CXCL1 | NM_001511 | Homo sapiens chemokine (C-X-C motif) ligand 1 (melanoma growth stimulating activity, alpha) | 299 | 0.003348 |
| CXCL12 | NM_199168 | Homo sapiens chemokine (C-X-C motif) ligand 12 | 5290 | 0.059292 |
| CXCL12 | NM_000609 | Homo sapiens chemokine (C-X-C motif) ligand 12 | 328 | 0.003671 |
| CXCL12 | NM_001033886 | Homo sapiens chemokine (C-X-C motif) ligand 12 | 773 | 0.008669 |
| CXCL14 | NM_004887 | Homo sapiens chemokine (C-X-C motif) ligand 14 | 3626 | 0.040636 |
| CXCL2 | NM_002089 | Homo sapiens chemokine (C-X-C motif) ligand 2 | 187 | 0.002097 |
| CXCL2 | NM_002089 | Homo sapiens chemokine (C-X-C motif) ligand 2 | 174 | 0.001950 |
| CXCL5 | NM_002994 | Homo sapiens chemokine (C-X-C motif) ligand 5 | 412 | 0.004616 |
| CXCR3 | NM_001142797 | Homo sapiens chemokine (C-X-C motif) receptor 3 | 110 | 0.001231 |
| CXCR7 | NM_020311 | Homo sapiens chemokine (C-X-C motif) receptor 7 | 4450 | 0.049879 |
| FAM19A5 | NM_015381 | Homo sapiens family with sequence similarity 19 (chemokine (C-C motif)-like), member A5 | 472 | 0.005288 |
| **Stem-Cell Renewal Markers** | | | | |
| SOX11 | NM_003108 | Homo sapiens SRY (sex determining region Y)-box 11 | 374 | 0.004195471 |
| SOX13 | NM_005686 | Homo sapiens SRY (sex determining region Y)-box 13 | 620 | 0.006952352 |
| SOX4 | NM_003107 | Homo sapiens SRY (sex determining region Y)-box 4 | 147 | 0.001650322 |
| SOX8 | NM_014587 | Homo sapiens SRY (sex determining region Y)-box 8 | 168 | 0.001886957 |
| SOX9 | NM_000346 | Homo sapiens SRY (sex determining region Y)-box 9 | 157 | 0.001764 |
| **Non-Muscle and Muscle Markers** | | | | |
| MEF2C | NM_002397 | Homo sapiens myocyte enhancer factor 2C | 185 | 0.0020712 |
| TNNT1 | NM_003283 | Homo sapiens troponin T type 1 (skeletal, slow) | 251 | 0.0028079 |
| TNNT2 | NM_000364 | Homo sapiens troponin T type 2 (cardiac) | 135 | 0.0015159 |
| DES | NM_001927 | Homo sapiens desmin | 8119 | 0.0910025 |
| DMD | NM_004010 | Homo sapiens dystrophin | 272 | 0.0030487 |
| MYO1B | NM_012223 | Homo sapiens myosin IB | 4117 | 0.0461424 |
| MYO1B | NM_012223 | Homo sapiens myosin IB | 572 | 0.0064137 |
| MYO1C | NM_033375 | Homo sapiens myosin IC | 33956 | 0.3805799 |
| MYO1D | NM_015194 | Homo sapiens myosin ID | 168 | 0.0018873 |
| MYO1E | NM_004998 | Homo sapiens myosin IE | 4950 | 0.0554848 |
| MYO9A | NM_006901 | Homo sapiens myosin IXA | 1093 | 0.0122483 |
| MYO9B | NM_004145 | Homo sapiens myosin IXB | 2468 | 0.0276586 |
| MYLK | NM_053025 | Homo sapiens myosin light chain kinase | 12346 | 0.1383769 |
| MYLK | NM_053025 | Homo sapiens myosin light chain kinase | 18846 | 0.2112306 |
| MPRIP | NM_015134 | Homo sapiens myosin phosphatase Rho interacting protein | 5293 | 0.0593245 |
| MPRIP | NM_015134 | Homo sapiens myosin phosphatase Rho interacting protein | 438 | 0.0049048 |
| MYLIP | NM_013262 | Homo sapiens myosin regulatory light chain interacting protein | 255 | 0.0028531 |
| MYO5A | NM_000259 | Homo sapiens myosin VA (heavy chain 12, myoxin) | 940 | 0.010534 |
| MYO6 | NM_004999 | Homo sapiens myosin VI | 288 | 0.0032327 |
| MYO10 | NM_012334 | Homo sapiens myosin X | 426 | 0.0047726 |
| MYO19 | NM_025109 | Homo sapiens myosin XIX | 2515 | 0.0281884 |
| MYO19 | NM_001033580 | Homo sapiens myosin XIX | 510 | 0.0057108 |
| MYO18A | NM_078471 | Homo sapiens myosin XVIIIA | 698 | 0.0078183 |
| MYO18A | NM_078471 | Homo sapiens myosin XVIIIA | 334 | 0.0037458 |
| MYH10 | NM_005964 | Homo sapiens myosin, heavy chain 10, non-muscle | 1736 | 0.0194521 |
| MYH11 | NM_001040114 | Homo sapiens myosin, heavy chain 11, smooth muscle | 413 | 0.004629 |
| MYH11 | NM_001040113 | Homo sapiens myosin, heavy chain 11, smooth muscle | 397 | 0.0044482 |
| MYH14 | NM_001077186 | Homo sapiens myosin, heavy chain 14, non-muscle | 1515 | 0.016983 |
| MYH9 | NM_002473 | Homo sapiens myosin, heavy chain 9, non-muscle | 15884 | 0.1780288 |
| MYH9 | NM_002473 | Homo sapiens myosin, heavy chain 9, non-muscle | 639 | 0.0071629 |
| MYL12A | NM_006471 | Homo sapiens myosin, light chain 12A, regulatory, non-sarcomeric | 45011 | 0.5044894 |
| MYL12B | NM_033546 | Homo sapiens myosin, light chain 12B, regulatory | 44829 | 0.5024526 |
| MYL12B | NM_033546 | Homo sapiens myosin, light chain 12B, regulatory | 31298 | 0.3507876 |
| MYL2 | NM_000432 | Homo sapiens myosin, light chain 2, regulatory, cardiac, slow | 279 | 0.003131 |
| MYL5 | NM_002477 | Homo sapiens myosin, light chain 5, regulatory | 1122 | 0.012573 |
| MYL6 | NM_079423 | Homo sapiens myosin, light chain 6, alkali, smooth muscle and non-muscle | 51777 | 0.5803207 |
| MYL6B | NM_002475 | Homo sapiens myosin, light chain 6B, alkali, smooth muscle and non-muscle | 7184 | 0.0805219 |
| MYL9 | NM_181526 | Homo sapiens myosin, light chain 9, regulatory | 16592 | 0.1859632 |

^a^ The fold difference represents the ration of intensity of each gene hybridized with the RNA isolated from hADSC normalized to the intensity of GAPDH gene. Each array was processed in an identical manner and the number represents an average of triplicate experiments from three independent cell isolates. Each gene is demarcated by the Genebank accession number, the description of gene and the common name. Genes are group by their distinct functional categories.
